# Digital Discourse, Secondary Victimization, and Psychological Harm: Mixed-Methods Analysis of System Justification in the #MeToo Movement

**DOI:** 10.2196/75533

**Published:** 2026-04-09

**Authors:** Harsh Parekh, Shriya Thakkar, Paras Bhatt, Patricia Akello

**Affiliations:** 1 Paul H. Chook Department of Information Systems and Statistics, Zicklin School of Business, Baruch College, City University of New York, New York, NY, United States; 2 Department of Prevention and Community Health, Milken Institute School of Public Health, George Washington University, 950 New Hampshire Avenue, Washington DC, DC, 20052, United States, +1 (202) 994-7400; 3 Department of Information Systems, Supply Chain & Analytics, College of Business, The University of Alabama in Huntsville, Huntsville, AL, United States; 4 Department of MIS and Cybersecurity, College of Business, University of Montana, Missoula, MT, United States

**Keywords:** harassment, mental health, inequities, gender, mixed methods

## Abstract

**Background:**

The #MeToo movement, initiated in 2006 and amplified on social media in 2017, mobilized women worldwide to share experiences of sexual harassment and assault online. While the movement increased awareness, it also revealed deep social divisions in digital spaces. Supportive discussions promoted solidarity and healing, whereas antagonistic responses reinforced backlash and secondary victimization. In India, the Indian Entertainment Industry (IEI) became a focal point where survivors’ disclosures highlighted structural gender inequalities. These polarized reactions function as digital-health signals, reflecting stigma, psychosocial distress, and conditions that shape women’s safety and mental well-being. Examining these narratives as indicators of public health risk helps identify patterns of structural inequity and secondary mental health burdens among survivors.

**Objective:**

This study examined online discourse surrounding #MeToo to identify forms of system-justifying narratives on social media and to assess how #MeTooIndia exposed institutional inequities within the IEI.

**Methods:**

This mixed-methods study comprised 2 components. In study 1, natural language processing was applied to analyze global #MeToo Twitter (subsequently rebranded X) discourse. From an initial corpus of 350,000 tweets, 205,082 were preprocessed, and sentiment and stance detection analysis identified 18,416 tweets expressing negative attitudes toward the movement. Latent Dirichlet allocation topic modeling extracted 22 topics, 12 of which aligned with system-justification categories, revealing distinct lexical and semantic patterns related to gender, institutional, and power dynamics. Two trained coders manually annotated a subsample to ensure conceptual clarity and interrater reliability. Study 2 involved qualitative, semistructured interviews with 20 academic experts in film, gender, and media studies to gather opinions on how #MeTooIndia influenced institutional discourse in the IEI and how these dynamics translate into digital and mental health risks.

**Results:**

Analysis of #MeToo Twitter discourse in study 1 identified 4 primary forms of system justification: by gender, by the institutional system, by backlash, and by victim-blaming. Gender and institutional system justifications were the most prevalent. Study 2 reinforced these findings, revealing how experts perceived #MeTooIndia as both empowering and constrained by entrenched institutional and cultural barriers. Together, our findings highlight the dual function of social media in promoting collective advocacy while reproducing conditions linked to gender-based violence, psychological stress, and reduced help-seeking—key digital and mental health concerns.

**Conclusions:**

This mixed-methods study reveals that digital discourse surrounding #MeToo often sustains existing gender and institutional hierarchies rather than dismantling them. Across Twitter data and expert interviews, gender and institutional system justifications emerged as dominant narratives, highlighting how online spaces can reinforce structural inequities while appearing progressive. Although #MeToo amplified visibility and awareness, its potential for lasting institutional change remains limited. These findings underscore the need for trauma-informed digital governance, public health recognition of online hostility as a psychosocial risk, and frameworks that situate digital activism to institutional reforms that support safety and mental well-being.

## Introduction

### Background

Initially founded by the New York-based social activist Tarana Burke in 2006, #MeToo aimed to empower women and support victims of sexual assaults in the United States. A decade later, the movement gained worldwide attention when actress Alyssa Milano popularized #MeToo with a viral Twitter (subsequently rebranded X) trend. The movement broadened awareness while reflecting the emotional strain tied to large-scale public disclosure [1,2]. From a public health perspective, this increase represents more than social awareness. It reflects large-scale digital expressions of trauma that reveal population-level exposure to gender-based violence (GBV) and related mental health consequences [3-5].

Gender equality movements such as #MeToo are often viewed as women-led initiatives [6], which shapes how people engage with them online. This framing also explains why public responses became sharply divided or polarized. Expressions of solidarity promoted healing and collective efficacy [5,7,8], while hostile or dismissive comments reproduced stigma and secondary victimization [9]. Prior analyses have shown that discussions around #MeToo contained both destructive elements, such as ridicule, rape references, and scandal language, and constructive ones emphasizing education, leadership, and rights [5,9]. Such polarization is health-relevant because antagonistic discourse can intensify psychological distress, discourage help-seeking, and perpetuate unsafe disclosure climates for survivors [3,10-12]. Opponents of #MeToo viewed the movement as threatening social identity and existing hierarchies [13,14], further sustaining online environments that can undermine survivor well-being and collective healing.

In this context, while the #MeToo movement resonated worldwide, its impact on the Indian Entertainment Industry (IEI), particularly Bollywood, was notably profound, serving as a catalyst that broke long-standing barriers of silence within the entertainment sector [15]. Within the IEI, disclosures emerged rapidly and publicly, revealing deep-seated gender hierarchies and power asymmetries that had long silenced survivors. For instance, late Bollywood choreographer Saroj Khan remarked, “Bollywood provides bread, at least doesn’t abandon women after rape” in the wake of the #MeToo movement in India [16]. Such comments exemplify how public figures can normalize or excuse sexual violence, reflecting the cultural narratives that sustain institutional denial and perpetuate psychological harm for survivors [17]. These justifications and dismissive reactions have measurable health implications. Studies show that negative or invalidating responses to #MeToo disclosures are associated with increased posttraumatic stress, anxiety, and feelings of isolation among survivors [18,19].

In the Indian context, such backlash often manifests through public shaming, social exclusion, and reputational damage, creating conditions of secondary victimization that discourage reporting [17,20,21]. Although social media facilitates visibility and mobilization, it also amplifies exposure to stigma and retraumatization, turning disclosure spaces into sites of both empowerment and psychological risk [1]. Thus, our analysis addresses calls to understand the system-justification narratives within #MeToo that reproduce structural inequities and generate secondary mental health burdens for survivors [9,19,22].

Alongside social and moral support, #MeTooIndia also generated negative reactions in the form of backlash, victim blaming, and social system justification [23]. In patriarchal contexts, women survivors often face reputational risks and moral scrutiny that discourage disclosure and silence voices of resistance [20,24]. Backlash also stemmed from a perception that #MeTooIndia threatened group identity and the existing social order, prompting many to defend traditional hierarchies through system-justifying narratives [25,26]. Previous research on #MeToo links disbelief and ridicule following disclosure to trauma recurrence, anxiety, and avoidance behaviors [9,10,22]. This study examines online reactions to #MeToo through the lens of a digital health discourse. Social-media discourse surrounding GBV functions not only as a forum for advocacy but also as a public-health signal that reflects collective trauma and emotional distress [4,18]. Such digital expressions can inform health care providers, mental health professionals, and public health practitioners about emerging patterns of psychosocial stress and the need for trauma-informed care and community-level interventions [27,28].

We treat the justification narratives surrounding #MeToo as infoveillance indicators, which are digital signals of GBV and secondary victimization addressing limitations identified in prior research [5,29]. Hostile or invalidating comments constitute secondary victimization [30,31], which research links to increased posttraumatic stress, anxiety, and social withdrawal among survivors [9,11,19,22]. Within patriarchal or celebrity-dominated contexts such as India’s entertainment industry, such backlash can reinforce stigma and reproduce structural inequities that exacerbate mental health burden [17,20]. Positioning the analysis within this digital-health framing enables examination of how social-media environments impact both public-health awareness and secondary psychological harm following GBV disclosure [3].

### This Study

#### Overview

Victim blaming stands as a key factor deterring women from coming forward or identifying their aggressors [5,32-34]. Building on this context, the current study focuses on entrenched gender hierarchies, institutional status quo, pushback on social movement, and victim-blaming as mechanisms of social system justification within the #MeToo movement. In extending prior work, our study moves beyond the initial disclosure phase to examine how system-justifying narratives evolve as digital signals of GBV [4,29,35,36]. Study 1 conducts a large-scale text mining analysis of Twitter data to identify and categorize system-justifying narratives that emerged during the #MeToo movement. Study 2 complements this with qualitative interviews focused on the IEI, offering deeper insights into how these narratives manifest within a specific sociocultural and institutional setting. Together, these studies offer a mixed-methods perspective on how digital backlash and justification discourse operate as online expressions of GBV, with implications for mental health and digital well-being.

#### Objectives

The objectives of this study were to (1) identify the forms of system-justifying discourse that emerged on social media during the #MeToo movement and (2) examine how #MeTooIndia revealed its effects on institutional systems within the IEI.

### Conceptualization: Social Media as a System Justifier

Social conditioning often creates circumstances in which seeking justice can, unfortunately, intensify the harassment experienced by victims [5,22,37]. Secondary victimization occurs through social media when the perpetrators face legal problems, and the community unjustly holds the victim responsible [38,39]. System justification theory suggests that “there is a general ideological motive to justify the existing social order,” and this motive is responsible for the “internalization of inferiority among the members of the disadvantaged group” [40]. System justification theory has been applied across disciplines, including public health, using established scales to assess how individuals endorse or legitimize existing social systems [41,42]. An example of a belief measure is the view that society is structured so that people generally get what they deserve. It is important to distinguish psychological system justification, which reflects unconscious cognitive motives to legitimize existing hierarchies, from strategic or systemic resistance, which involves deliberate actions by individuals or institutions to preserve power and resist reform [43,44].

System justifiers create a fear of judgment or retaliation for sexual victims, drastically reducing the disclosure [19,45,46]. A substantial number of tweets criticized individuals, including prominent figures, who had blamed victims of sexual harassment for their experiences, thereby undermining the feminist movement [22,47]. The inclination to rationalize current social structures may be particularly strong in the context of gender dynamics compared with other intergroup settings, such as racial, religious, or ethnic groups, as gender relations are deeply interdependent within social and economic systems [25]. The types of system justifications are listed in Table 1.

**Table 1.** Type of system justifications.

Justification type	Definition
Gender system justification	The “extent one accepts gender hierarchy as legitimate” (page 1). This includes upholding traditional gender roles, norms, and power dynamics within the system [48].
Institutional system justification	This type of system justification focuses on the “legitimation of institutions and authorities, denial or minimization of system problems or shortcomings” (page 881). It involves defending workplaces, industries, legal systems, or social hierarchies [40].
Victim-blaming system justification	This type of justification is known as the derogation effect. It indicates that “victim is deserving of the injustice and responsible for his/her own misfortune” (page 5912). This behavior often involves questioning the credibility of the victim, scrutinizing their agency, and fabricating their experiences [39].
Backlash system justification	This type of justification indicates “vociferous and frequently violent opposition” toward “any actual or potential gains made by disadvantaged groups” (page 133-134). This behavior includes disapproving of facts and studies recognizing the prevalence of sexual harassment, highlighting the threat to individual freedom, and sympathizing with defamation received by the accused [49].

Similarly, investigating the 2017 Women’s March movement, researchers found that mostly in the case of men (who identify gender as less important to one’s self-concept), exposure to gender movement increased system justification tendency [48]. Furthermore, men who expressed support for gender system justification believed that #MeToo unjustly tarnished the reputation of men. Evidence indicates that the endorsement of sexist ideologies is connected to individuals’ perspectives on sexual harassment and their stance toward the #MeToo movement [50].

While defending the existing gender system may represent the main narrative for system justification within such movements, it is not the only type represented on social media. Reports have highlighted the limited accountability of businesses and institutions during the #MeToo movement [51]. Sociocultural and organizational systems often reinforce beliefs and behaviors that perpetuate sexual harassment and misconduct. Furthermore, victims bear the personal cost of legal representation, while institutions support the defense of powerful offenders. Such dynamics contribute to secondary victimization and institutional retaliation, maintained through system-justifying mechanisms [52].

Witnessing online victimization has been shown to promote dehumanization of victims, leading to perceptions that they are less deserving of respect or protection [21,53]. Such cognitive and emotional distancing contributes to harmful social norms that normalize violence and reduce collective empathy, which are both recognized as public health concerns [10,19]. Victim-blaming justifications are produced when individuals experience identity threats and defensively protect their self-image by partially or fully attributing blame to the victim [5,18]. These justifications are reinforced by gender-powered dynamics and male sexual aggression, which in turn increase women’s vulnerability and sustain beliefs rationalizing the just world hypothesis [53-55].

Victim-blaming justifications not only shift attribution away from perpetrators but also contribute to resistance against the broader goals of the #MeToo movement. This form of discourse often frames the movement as an overreaction or a threat to individual freedom, thereby undermining efforts to address GBV and promote safer social environments [5,17]. Such responses reflect a broader tendency to preserve positive perceptions of existing social systems, even when these systems sustain inequities that have significant public health implications for victims’ mental and physical well-being [19,29,49]. Earlier research indicates that the movement could provoke even more intense negative responses from those who are unsympathetic or hold contrary beliefs about its objectives [5,36,48].

Understanding how systems justify and reproduce themselves is key to identifying the social and psychological barriers that impede collective action. From a public health perspective, such insights can guide targeted strategies to disrupt these barriers, strengthen community engagement, and sustain online activism that promotes equity and safety.

## Methods

### Study Design

The global #MeToo movement revealed significant regional variation in engagement and public discourse [35,56]. Recognizing that social movements addressing GBV have broad implications for population health and social well-being, we adopted a mixed-methods design to examine how digital narratives reinforce or challenge systemic inequities. This study comprised two components: (1) a quantitative analysis of tweets to assess the global diffusion and framing of #MeToo via social media, and (2) a qualitative study using in-depth interviews focused on #MeTooIndia to contextualize the movement’s local impacts.

This research used a sequential explanatory mixed-methods design in which study 1 directly informed study 2 [57]. The quantitative analysis of Twitter discourse identified 4 dominant forms of system justification, and these empirically derived categories guided the development of the semistructured interview guide (Multimedia Appendix 1). Study 2 was therefore designed to contextualize, elaborate, and extend the patterns observed in study 1 by examining how similar justification mechanisms were understood within the localized context of the IEI. During interpretation, findings from both studies were integrated to provide a consolidated understanding of how digital narratives and institutional responses interact during #MeToo.

In the first study, we collected tweets without geographic restrictions to minimize sampling bias and capture cross-cultural expressions of system justification. Regional and cultural contexts can shape how individuals legitimize or contest social hierarchies online, influencing public perceptions of gender justice and safety—key determinants of health equity. Guided by system justification theory, which posits that individuals tend to rationalize and defend existing social structures even when they sustain inequality, we used natural language processing–based topic classification to identify patterns of justification within global #MeToo discussions. We then complemented these findings with an in-depth case study of #MeTooIndia, drawing on expert interviews to explore how these justifications manifest within the Indian sociocultural and institutional context, which led to our second study.

In our second study, we examined the social and behavioral consequences of the #MeTooIndia movement within the context of IEI. Prior research underscores that diverse cultural settings can obscure interpretation unless analyzed within a specific context [58]. IEI, particularly Bollywood, offered a compelling case as the movement gained strong momentum, generated widespread public engagement, and empowered women actors to voice experiences of sexual misconduct. To capture these dynamics, we conducted interviews with research scholars specializing in media or film studies to gain nuanced insight into the social and institutional responses surrounding #MeTooIndia.

While the 2 studies used different methods, they collectively offer a multidimensional understanding of digital activism and its societal implications. The natural language processing–based analysis in study 1 captures large-scale public discourse and patterns of system justification, whereas the expert interviews in study 2 provide contextual depth and interpretation of those narratives. Together, these complementary approaches offer a more comprehensive view of how such digital feminist movements intersect with cultural norms, institutional accountability, and collective well-being. Consistent with Gorissen et al [22], who found that Twitter hosted 61.4% of sexual victimization disclosures during #MeToo, our focus on Twitter data ensured a consistent and extensive dataset for examining system-justifying narratives that influence public attitudes toward gender equity and safety.

### Transparency and Openness

**Figure 1.**
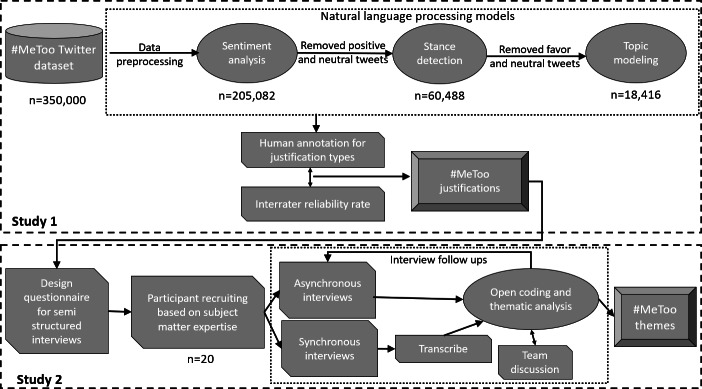
Overall methodological flow illustrating the mixed-methods design across study 1 and study 2.

The processed data supporting our conclusions, along with the Python (Python Software Foundation) syntax required for reproducing the results, can be obtained from the authors upon request. All analyses were performed using Python libraries. Please note that this study’s design and analysis were not preregistered because the research was exploratory and aimed at developing theoretical and methodological insights rather than testing predefined hypotheses (Figure 1).

### Ethical Considerations

For quantitative study 1, all data were obtained from publicly accessible sources on Twitter. Posts shared under the hashtags (#MeToo and #metoo) represent public communications intended for open audiences. No personal or identifying information about individual users (such as usernames, user IDs, or direct mentions) was retained, as all data were deidentified before analysis. All analyses were conducted on aggregated text data, focusing on linguistic and thematic patterns rather than individual users or specific tweets. Results are therefore presented in group form to ensure that no inferences can be made about any specific user. Given ongoing concerns about the potential for reidentification of social media users, even when direct identifiers are removed, we took additional precautions when presenting illustrative material (sample tweets). All sample content used for explanation in this paper was paraphrased, and any original handles were replaced with neutral placeholders to prevent traceability. Finally, while we do not publicly share any of the analyzed data, we outline more details in the “Data Availability” section [59,60].

For study 2, all participants were experienced academics familiar with research ethics and the principles of informed consent and institutional review. Each received an information sheet describing this study’s objectives, procedures, and data-use policy. Participation was voluntary. Participants were interviewed strictly in their professional capacity, and no identifiable personal data was collected. The authors analyzed qualitative materials that were recorded in a fully anonymized form. This study did not involve interaction or intervention beyond the collection of anonymous responses. Accordingly, the work met criteria for minimal-risk social research and did not constitute human participants research requiring institutional review board oversight under the US Common Rule (45 CFR §46.102) [61]. Additionally, the research adhered to the ethical principles of the Declaration of Helsinki [62] and the World Health Organization’s (2001) guidelines for ethical qualitative inquiry [63].

To protect privacy and confidentiality, all interviews were conducted and recorded in English with explicit participant consent. Transcripts were deidentified, assigned numeric codes, and stored on password-protected devices accessible only to the research team. No identifiable information, images, or supplementary materials are included in this paper. Participation was entirely voluntary, and no monetary compensation was provided. Table 2 summarizes the demographic characteristics of the scholars and experts who participated in this study.

**Table 2.** Demographic profile of scholars and experts interviewed for study 2 (N=20). The names of the respondents referred to in this research are pseudonyms, and identities are kept strictly confidential to maintain anonymity. Professional or educational engagement and country of professional or educational attainment are as reported.

Name	Identified by the pronouns used	Current professional or educational engagement	Country of professional or educational affiliation
Daniel	He or his	Professor	United States
Smita	She or hers	Associate professor	United States
Sarita	She or hers	Associate professor	United States
Raya	She or hers	Professor	United States
Ramya	She or hers	Assistant professor	India
Irrfan	He or his	Associate professor	Turkey
Karanjeet	He or his	Assistant professor	India
Latika	She or hers	Doctoral candidate	United Kingdom
Nazreen	She or hers	Doctoral candidate	United States
Naved	He or his	Doctoral candidate	Turkey
Mita	She or hers	Assistant professor	United States
Neeta	She or hers	Graduate student	United States
Kirti	She or hers	Assistant professor	India
Kamal	She or hers	Assistant professor	United States
Rahul	He or his	Doctoral candidate	United States
Smitra	She or hers	Graduate student	India
Tupur	She or hers	Doctoral candidate	India
Ipsita	She or hers	Graduate student	India
Ayanti	She or hers	Graduate student	India
Jincy	She or hers	Doctoral candidate	India

### Study 1: Quantitative Twitter Analysis

#### Study Design and Data Collection

We used publicly accessible tweets as the main source of our data for conducting a topic-wise classification. We used the Twitter API platform to extract tweets from #metoo and #MeToo for a period of 1 month from November 2017 to December 2017. The extraction resulted in a corpus of 350,000 tweets. This dataset provides a rich corpus for examining public discourse and sentiment around the movement, offering important insights into how individuals communicate experiences of GBV and solidarity in digital spaces. The data included several identifiers such as postdate, user description, and engagement metrics about the likes, retweets, and replies, allowing us to examine both message content and patterns of social amplification.

**Figure 2.**
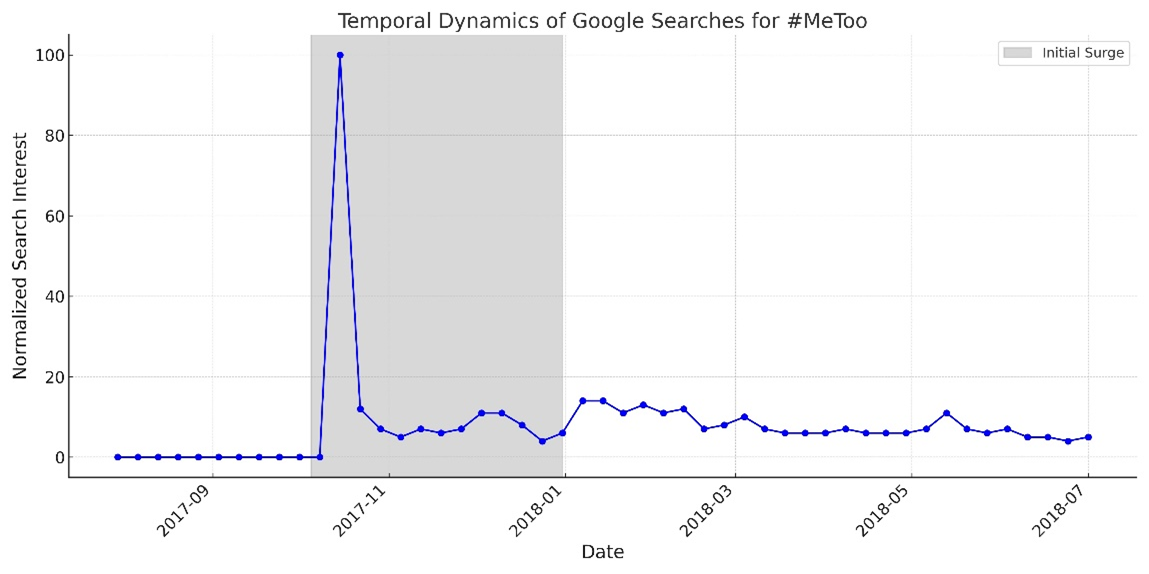
Temporal dynamics of Google search interest in #MeToo from November 2017 to December 2017.

The global #MeToo conversation began on October 15, 2017, when Alyssa Milano invited survivors of sexual harassment to share their stories publicly. The movement quickly transcended linguistic and cultural boundaries, inspiring localized expressions such as *#YoTambien* in Spanish-speaking countries, *#BalanceTonPorc* (“squeal on your pig”) in France, *#quellavoltache* in Italy, and *#RiceBunny* in China—a creative response to online censorship. We strategically collected the data after the first sporadic spike (which turned out to be the highest over time). Figure 2 shows Google Trends (Google LLC) in the #MeToo movement. As we wanted to capture various system justifications, the period after the first sporadic spike was deemed appropriate [22].

Textbox 1 showcases exemplar tweets illustrating the various justification types that serve to diminish the impact of the #MeToo movement. All tweets in Textbox 1 are paraphrased and deidentified to prevent traceability to individual users. Simple removal of identifiers is insufficient, as advanced fuzzy-matching and text-retrieval techniques can enable reidentification of social media posts. To protect user anonymity, we replaced all handles with neutral placeholders (eg, ABC, @DEF, and @GHI) and reworded examples while preserving their conceptual meaning.

Textbox 1. **Sample tweets for justification types.**

- Gender system justification: “we have to "go back" to giving men authority over women in the workplace, home & society in general so they will STOP taking advantage #MeToo.”
- Institutional system justification: “@ABC You've been in #Hollywood for 60 years, you've worked with, kept quiet about, helped to cover up, so why now?”
- Victim-blaming system justification: “@DEF Yeah it works in mysterious ways because she knew he was a sexual predator and still worked with him”
- Backlash system justification: “@GHI: Texas Attorney General's top aide mocks 'pathetic' #MeToo movement and calls women's marchers 'c*nts'”

#### Analysis

Preprocessing of the #MeToo Twitter dataset was essential to ensure data quality and analytical reliability (Multimedia Appendix 2). We streamlined the data by performing initial cleaning and normalization tasks, such as removal of URLs, hashtags, and user mentions, correction of typographical errors, and standardizing text to a uniform lower case, and then further refined our dataset by eliminating special characters, tokenizing text, and handling abbreviations. These comprehensive steps concentrated our analysis on 205,082 tweets, priming them for further analysis. In the sentiment analysis phase, we used the Python package *TextBlob* to perform sentiment detection on the subset of 205,082 tweets identified postinitial preprocessing.

This analysis classified the emotional tone of each tweet into positive, neutral, or negative categories. Subsequently, we refined our dataset by excluding tweets that exhibited positive and neutral sentiments. We drew from the work of Reyes-Menendez et al [64], which used n-grams analysis to reveal a dual framing of the #MeToo movement—one destructive, associated with terms such as cowards, rape, and scandals, and another constructive, linked to words such as educate, leader, and rights.

For our study of justification narratives, we focused on the former, harnessing sentiment analysis and stance detection techniques. This allowed us to refine our dataset to specifically include those tweets that aligned with destructive negative discourse, providing a pertinent sample for our analysis of justification cases. This filtration process resulted in a concentrated sample of 60,488 tweets, each expressing a negatively worded sentiment, which provided a more precise basis for examining the adverse reactions associated with the #MeToo movement. After filtering our dataset to include only the negatively worded tweets, we used the *NLTK* Python package for stance detection to categorize each tweet’s attitude toward the #MeToo movement as supportive, neutral (or factual), or opposing. We then removed tweets with supportive and neutral stances, resulting in a final dataset of 18,416 tweets that explicitly opposed or criticized the movement by justifying existing social norms, thereby focusing our analysis on expressions of dissent.

We implemented a 2-pronged content analysis strategy to examine the semantic structures within our tweet dataset. First, we used latent Dirichlet allocation topic modeling, which identified and extracted 22 distinct topics representing underlying themes in the discourse. Upon further analysis, we found that 10 of these topics were not directly aligned with any justification narratives, while the remaining 12 topics were systematically categorized into specific justification categories.

Each justification type exhibited distinct lexical and semantic signatures. From the latent Dirichlet allocation topic modeling results, we observed that the topics associated with each system justification type contained unique keyword structures indicative of the underlying sentiment. For instance, “backlash system justification” topics included words such as “movement,” “vicious,” and “sex,” hinting at an undercurrent of resistance to the #MeToo narrative. “Victim blaming justifications” were laced with terms such as “rape,” “society,” and “refuse,” reflecting accusatory and deflective tones. Meanwhile, “institutional justification” topics incorporated institutional and procedural terms such as “resign,” “misconduct,” and “allegation,” suggesting a focus on systemic and structural dimensions. Lastly, “gender system justification” topics distinctly featured “woman” and “man” in their discourse, emphasizing the gendered aspects of the conversation.

These keyword patterns are illustrative of the diverse justifications and sentiments present within the #MeToo-related discourse captured in our dataset. In the second phase, we complemented the computational results with human-coded annotation to refine the justification categories within our tweet sample. Specifically, we recruited 2 trained annotators to collaboratively analyze a representative set of 250 tweets, with the aim of establishing a shared conceptual framework for the various categories of justifications. This process was guided by detailed annotation protocols to ensure conceptual clarity, coding consistency, and interrater reliability.

The interrater reliability, measured using Cohen , was found to be 0.91, reflecting a high degree of agreement between the annotators and confirming the robustness of the annotation schema. With this validated framework, we proceeded to annotate a larger subset of 18,416 tweets. We used the insights gained from the initial 250 tweets to train a supervised machine learning model, thereby scaling our classification effort across the entire dataset. This model was designed to categorize tweets into 1 of 4 that justification types or into a fifth category indicating the absence of a discernible justification.

This process allowed us to accurately distribute our sample into proportionate classifications of each justification type, thus enabling a comprehensive and quantifiable analysis of the dominant narratives in the relevant tweets. Discrepancies between annotators were resolved through discussion and iterative refinement of the coding guidelines, resulting in strong interrater agreement and improved clarity in classification criteria. The precision of our classification framework established a robust foundation for subsequent analyses and interpretations of the data. Across the 18,416 negatively valenced tweets, institutional system justification was the most prevalent (7366/18,416, 40%), followed by gender system justification (6814/18,416, 37%), victim-blaming justification (2210/18,416, 12%), and backlash justification (2026/18,416, 11%).

### Study 2: Qualitative Thematic Analysis

#### Study Design and Recruitment

Study 2 builds upon the findings of study 1 by incorporating perspectives from academic experts in the field. We conducted 20 in-depth interviews with scholars and researchers specializing in gender, media, and film studies—particularly those focused on Hindi cinema and the Indian entertainment context. The objective was to understand how #MeTooIndia influenced discourse and practices within the IEI. In-depth interviews are a well-established qualitative method for obtaining detailed insights into participants’ beliefs, experiences, and interpretations of a given phenomenon [65-67]. We targeted academics and scholars because of their capacity to provide theoretically informed and relatively impartial reflections on the movement—an important complement to journalistic and activist perspectives that have dominated public narratives.

Using purposive sampling [68], the current study recruited professors, research scholars, and doctoral candidates affiliated with universities and research institutions across the United States, India, the United Kingdom, and the Middle East who specialized in media and film studies, with a focus on Indian cinema. Potential participants were identified through prior publications and conference presentations in feminist media studies, the IEI, social-media activism, and cinema or film studies. We emailed 80 eligible academics, describing this study’s objectives and inviting them to participate in an interview. Of these, 29 responded, and appointments were successfully scheduled with 20 (36%) participants. The remaining 9 (11%) participants did not provide availability. Ultimately, 20 (25%) participants of the invited pool completed this study and formed the final analytic sample.

Recruitment was performed through personalized email invitations sent directly to eligible participants, describing this study’s objectives, the voluntary nature of participation, and procedures for ensuring confidentiality. Those who agreed to participate provided written informed consent electronically before interviews. Data were collected between January and May 2021, a period shaped by the COVID-19 pandemic, which redefined research logistics and accelerated the adoption of virtual data-collection tools. We conducted 13 synchronous interviews via Zoom and 7 asynchronous interviews through email correspondence, allowing for flexibility in scheduling, geographic reach, and iterative follow-up questions when deeper elaboration was required. This mixed interview format provided both immediacy and reflection, aligning with evolving qualitative research practices during pandemic conditions [69-71].

#### Analysis

All interviews were transcribed verbatim and imported into NVivo 12 (QSR International) to facilitate systematic coding and data management. Analysis followed an inductive thematic approach using the constant comparison method [72]. Each transcript was reviewed line by line to identify recurring ideas, patterns, and contradictions both within and across cases. In the first coding cycle, each author independently performed open coding to generate preliminary labels grounded in the participants’ language. After multiple reviews of the transcripts, codes such as victim blaming, system, and institutional justification began to emerge. The second coding cycle involved axial coding, where similar concepts were grouped and relationships between categories were explored [73].

To enhance methodological rigor, intercoder reliability was assessed by double-coding a subset of transcripts and comparing agreement rates. Intercoder reliability is crucial as it showcases the methodological approach of achieving consensus in coding among the research team [74]. Discrepancies were resolved through discussion until consensus was reached, and a finalized codebook was developed. Once coding consistency was achieved, all transcripts were recoded using the agreed codebook, and thematic clustering was conducted within NVivo to synthesize findings. Analytical memos were maintained throughout to ensure transparency of interpretation and reflexivity among the research team.

## Results

### Study 1: Quantitative Study

Gender system justification emerged as the most prevalent category, representing 37% of the dataset and reflecting discourse that reinforces established gender hierarchies and traditional gender norms. Institutional system justification accounted for 40% (7366/18,416), characterized by narratives that defend organizational practices or minimize institutional accountability in cases of harassment and misconduct. Smaller yet meaningful proportions included backlash system justification (2026/18,416, 11%) and victim-blaming system justification (2210/18,416, 12%), illustrating persistent efforts to discredit the #MeToo movement or to shift responsibility onto victims. Together, these findings provide a quantitative overview of how online conversations reproduce societal power structures that sustain gender-based inequities—factors with significant implications for public health, safety, and psychosocial well-being.

The distribution captures the relative prevalence of each justification type, offering a data-driven perspective on the broader discourse surrounding gender justice and institutional accountability. Thus, study 1 offers a global quantitative map of justification narratives, while study 2 qualitatively examines these dynamics within #MeTooIndia in the IEI, illustrating how cultural norms and institutional structures shape experiences, responses, and barriers to gender equity and collective well-being.

### Study 2: Qualitative Data Collection

#### Overview

The final thematic analysis yielded 3 dominant themes (eg, gender system justification, victim blaming, and institutional system justification) reflecting the influence of #MeToo within the IEI, paralleling patterns identified in the quantitative Twitter analysis.

#### Gender System Justification

Despite #MeToo’s global aim to promote feminist awareness and counter sexual harassment, most participants observed little progress within the IEI, noting that existing gender hierarchies largely remain intact. This is an important concern for women’s digital and societal well-being, including their mental health and everyday safety, as entrenched gender norms shape vulnerability to harassment and constrain pathways to speak up or seek support. According to Daniel, a media professor from the Pacific West, United States:

*I think the #MeToo movement added some urgency to an already-ongoing set of changes, which I would characterize as an evolution rather than a revolution. Yes, there has been more diversity in representations of women, more women characters who exert greater autonomy and mastery, but there is still a tendency to show traditional kinds of stereotypical femininity as worthy of reward.*

While Daniel highlighted the persistence of gendered portrayals, Smita, a professor of cinema and photography of Indian American descent from the Midwest, elaborated on the deeper structural inequities within the industry:

It has brought attention to sexual exploitation within the industry, made public what insiders knew—but I would not go so far as to say, it has revolutionized anything. There is a great deal of power inequality in the industry; a lot of insecurity produced by having to stay on the right side of those in power (monopoly of a few big names); networks are brutal. Without any of these changes, things will not change much.

Several notable figures in the IEI attributed the ongoing challenges not only to the industry but also to the broader social system. For instance, Bollywood producer Vinta Nanda expressed that this issue is “not a Bollywood habit but a very Indian habit” [75]. Echoing this sentiment, Latika, a doctoral candidate pursuing her PhD in the United Kingdom, blamed the current political party in power, stating that:

#MeToo hasn’t been able to revolutionize Bollywood, not to the extent needed, but then India is currently under an unprecedented repressive and gender-violent religious fundamentalist regime, which acerbates the problem.

Irrfan, a professor of media and communications from the Midwest United States, offered a structural critique of #MeToo:

Social movements that are primarily organized by online campaigning often capture public attention quickly. Online users could effectively bring light to social injustice and accumulate public support in a short period. However, such organic and leaderless movements often do not have sufficient organizational capabilities to challenge the existing gender system and keep pushing for changes that could potentially challenge our society’s power-relations.

Irrfan highlighted a central limitation of #MeToo as an “organic” and “leaderless” movement, which has struggled to confront the gendered power dynamics within India’s sociostructural context. Scholars argue that the movement has largely amplified the voices of a privileged minority while neglecting those of marginalized and precariously employed women [76]. Others suggest that online social movements promote broader engagement than traditional offline activism, as digital platforms enable individualized participation and storytelling in place of hierarchical leader-driven conventional movements [77]. Additionally, the IEI is not governed by formal workplace policies or oversight mechanisms comparable to other sectors, leaving issues of women’s safety and protection as weakly regulated and contributing to increased mental health concerns. Overall, the expert testimonies argue for treating GBV in the IEI as occupational and digital health concerns that require structural interventions: standardized industry policies, independent redress mechanisms, survivor-centered mental health services, and safer digital reporting infrastructures. They also highlight the need for coordinated platform-industry-state action to ensure that the visibility generated by movements such as #MeToo leads to durable protections for women victims, rather than remaining confined to short-lived, elite-driven online attention.

#### Victim Blaming System Justification

Although #MeToo gained visibility in India, women victims who disclosed experiences of harassment frequently encountered backlash and blame from influential figures seeking to silence their voices. Similar to the #SurvivorPrivilege hashtag movement in 2014, Indian media narratives during #MeToo reinforced victim-blaming discourse, depicting survivors as manipulative or vindictive actors intent on falsely accusing men [9]. A widely publicized example involved actress Tanushree Dutta, who relocated to the United States after facing intense public hostility and media scrutiny following her #MeToo disclosure. Experts noted that these digital reactions operate as forms of secondary victimization, generating emotional strain and shaping whether women feel safe seeking support or speaking publicly. When asked about the movement’s effectiveness in confronting India’s long-standing culture of victim blaming, Daniel commented:

As long as victim blaming and victim shaming still get treated seriously in the press (and the courts), my answer to that question is, it has not gone far enough. Those who have been abused and exploited need to continue to speak up; witnesses need to be spoken up; bystanders need to be ready to believe uncomfortable things about people they know. It is worth noting that this need not be malicious to be problematic—some men who are not themselves abusers are likely trying to make sense of the idea that men with whom they have worked, their friends and role models, potentially, have engaged in immoral behavior. They try to resolve that uncomfortable inconsistency between what they think they know and what they’re hearing by rejecting the new information.

Daniel emphasized that countering victim-blaming requires empowering women to share their stories openly and fostering conversations that challenge victim shaming. He highlighted the need to expose offenders, create safe spaces for women to speak about their experiences without fear, and encourage bystanders to confront the possibility that individuals within their professional networks could be perpetrators. These goals also extend to digital health and safety, given that survivors’ willingness to disclose often depends on how platforms handle backlash, disbelief, and reputational risk.

In contrast, Sarita offered a more nuanced perspective, acknowledging both the limitations and potential of the #MeToo movement in challenging victim-blaming norms:

One of these tactics in Bollywood—a hotbed of the neoliberal beauty-fashion complex—has been to discredit these women on the basis of beauty norms. It becomes effective in a world mired in the logic of fashion as social capital. This form of slut-shaming, victim-blaming misogyny is effective in Bollywood to some extent. Yet what was new about #MeToo was that unlike earlier, (for instance, in the Shiney Ahuja case, who was arrested in 2009 for raping his then 19-y-old domestic help), there was a spotlight on the narrative of the victim. #MeToo Bollywood became her story. The narrative emphasis placed more screen time on the victim/survivor rather than the harasser. This is new, it helped that the women were also not dehumanized: it helped that we knew them through film roles earlier.

Sarita’s reflection highlighted an important shift in how survivor narratives were framed—away from silencing and toward recognition and visibility. This shift, she believed, has the potential to reduce stigma, support mental well-being, and strengthen collective efficacy among women by validating personal experiences and encouraging shared storytelling. Changing collective narratives around women’s stigmatized experiences has been shown to foster online connection, mutual validation, and collective identity formation, all of which can help challenge misogynistic discourse and improve mental health [78]. Smita, another participant, noted that this collective storytelling offered a new form of solidarity:

By naming the perpetrators, the campaign made formal what was a circle of solidarity amongst women—what was previously whispered was now made public so women could be warned. To me this was the most useful aspect of the campaign. Now, this is not the same as going to court, naming the perpetrator, giving evidence, a process that takes its toll in terms of years and other costs. So, it is possible that #MeToo may end up diluting the victim’s voice, the charge is that anyone can say whatever, etc. It is possible, but I think for the moment it has succeeded in sounding a warning note to potential perps. However, in the long run, as long as the severe imbalance remains in the industry, this campaign too may lose its credibility and power.

Thus, despite the limitations, participants viewed #MeToo as a powerful, if temporary, mechanism for achieving symbolic and restorative justice through visibility, solidarity, and public accountability. Although some scholars argue that these rapid forms of justice may be transient and fail to address deeper structural determinants of sexual misconduct [79,80], the movement nonetheless represents an essential step toward disrupting the silence around GBV. Taken together, these insights position victim-blaming and digital backlash as both digital and public health concerns, shaping survivors’ emotional well-being, safety, and ability to access redress within and beyond digital spaces.

#### Institutional System Justification

A striking contradiction revealed by the #MeToo movement in the IEI was the exposure of several acclaimed filmmakers, directors, and actors who were simultaneously celebrated for women-centric films and public advocacy for gender equality, yet accused of sexual harassment by colleagues in the industry. This pattern echoed high-profile cases in the United States involving R Kelly, Harvey Weinstein, and Bill Cosby, drawing similar attention to India among celebrity figures such as Subhash Ghai and Vikas Bahl (filmmakers), Alok Nath (actor), and Varun Grover (writer-comedian). While the allegations sent shockwaves through the public, many cases were quietly dismissed or settled, raising questions about institutional accountability and the fragility of justice when social status and reputation are at stake. Raya, a media professor in the United States, reflected on the broader cultural logic that enables such contradictions:

*This does not surprise me at all. There is a self-declared distance Bollywood or Indian cinema directors have often maintained between “art” and “life.” Representations of the female body and female sexuality have traditionally always been the prerogative of men in most cultures. Along with that, there has never been any mandate or prohibition against commodification or exploitation of the same body or femininity. In India, where there’s also such a strong social binary between the public and the private woman—the actress and the wife—in terms of sexual politics, such an assumption of entitlement regarding “public women” or actresses must come quite easily to men in power, whether they are artistic, creative, or not. There may also be that “great men” syndrome whereby many of them believe that as artistes they are not subject to the norms of what is called “bourgeois” morality by the avant-garde in India.*

Raya’s observation underscores how creative industries often justify misconduct through artistic exceptionalism—an attitude that normalizes exploitation as an accepted by-product of artistic freedom. Such institutional rationalizations mirror system-justifying processes that protect reputational hierarchies and maintain power asymmetries. Daniel and several other participants discussed how this contradiction extends to the production of women-centric films. As Daniel noted:

*The film industry is, at its core, an industry. Directors and producers are artists, certainly, but their eye—and the eye of their investors and funders—is on the bottom line. If they believe there is a market for a female-centric movie, they will make it even if it is not entirely consonant with their own personal beliefs and attitudes. It’s also important to remember that acting in ways inconsistent with sincerely held beliefs is hardly unusual.*

Collectively, participants highlighted the gap between gender-progressive representations on screen and persistent inequities off-screen. They pointed to entrenched gender ideologies, victim-blaming tendencies, and the commodification of women’s bodies as markers of an institutional culture that simultaneously markets empowerment and reproduces exploitation. Several experts observed that the performative nature of the industry—where acting and pretense are core to professional success—creates conditions in which abusers maintain a convincing public façade, reinforcing impunity.

These narratives show how social structures that normalize gendered entitlement and discourage reporting can generate psychological strain, career marginalization, and reduced well-being among women. Addressing institutional system justification in such creative settings requires more than media accountability; it demands organizational and occupational frameworks that prioritize equity, enforce safeguards against harassment, and cultivate supportive environments for women survivors. From a public health lens, the IEI’s gender inequities constitute a preventable occupational health crisis requiring multilevel interventions. For instance, digital health technologies offer scalable solutions for reporting, prevention, mental health support, and accountability—bridging the gap between performative empowerment and substantive safety. By combining public health frameworks with digital innovation, the industry can move beyond performative allyship toward measurable, sustainable gender justice that protects both mental well-being and professional equity.

## Discussion

### Social Media System Justification on Twitter

This study examined how social media discourse surrounding the #MeToo movement reflects and reproduces system-justifying narratives related to GBV. Consistent with this study’s objectives, the analysis identified 4 dominant forms of system justification—gender, institutional, victim-blaming, and backlash—across a large corpus of Twitter data and expert interviews. Study 1 demonstrated that online conversations frequently defended existing gender norms and institutional structures, with gender and institutional justification emerging as the most prevalent categories. Such defense mechanisms reflect social conditioning, resistance to change, and selective exposure to congruent beliefs online [81,82].

Study 2 further contextualized these patterns within the IEI, revealing that even as #MeTooIndia expanded public dialogue on sexual harassment—entrenched hierarchies, reputational risks, and institutional inertia continued to constrain meaningful reform. Together, these findings indicate that while the #MeToo movement fostered visibility and collective awareness, digital spaces also became arenas where system-justifying discourse reinforced social inequities and psychological strain, consistent with prior work showing that exposure to gender movements can amplify defensive reactions [48,50].

From a public health standpoint, these polarization patterns have psychosocial implications. Online gender-justifying narratives perpetuate stigma, discourage disclosure of sexual harassment, and increase survivors’ emotional distress [10,19]. The normalization of inequitable discourse can further sustain hostile digital climates that contribute to secondary victimization—a public health concern linked to trauma recurrence and barriers to help-seeking [22,38]. The high prevalence of institutional justification observed in the dataset also reflects persistent trust in existing social and institutional structures, even when those systems perpetuate inequality [20,21,29]. System justification fulfills a psychological need to defend and justify existing social arrangements, even if those are disadvantageous to society, as it is often perceived as psychologically comforting to individuals [40].

Institutional justification in feminist movements often works to preserve the status quo, especially when current arrangements benefit those in power [36,49]. These dynamics help explain why movements such as #MeToo can expose systemic issues and yet still fall short of durable institutional change. Finally, victim blaming and backlash justifications appeared less frequently but remained consequential. While victim-blaming and backlash in feminist movements have been widely examined [21,29,32,83,84], our findings show a comparatively lower prevalence of such narratives in the #MeToo discourse on social media. The wide circulation of survivor stories helped render overt victim-blaming less acceptable, reshaping public perceptions of sexual harassment and assault [5,33]. However, amplifying women’s voices through #MeToo also led some men to perceive themselves as unfairly victimized, fueling backlash that reasserted traditional gender hierarchies [20,85].

The “#MeTooLate Effect,” where delayed disclosures prompted disbelief or blame, underscores persistent biases that privilege certain forms of reporting [21,54]. Perceived credibility often follows a hierarchy shaped by social expectations of an “ideal” victim (such as immediate reporting or corroborating witnesses), thereby marginalizing survivors who do not conform to these norms [11,86]. Overall, these findings suggest that while social media amplifies awareness of GBV, it also functions as a mirror of societal polarization. This simultaneously promotes empowerment and entrenches opposition. Thus, digital backlash can increase psychological distress, inhibit help-seeking, and perpetuate stigma surrounding GBV [3,11,19]. Addressing these online narratives through trauma-informed communication, digital moderation, and survivor-centered discourse strategies is essential for promoting mental well-being and social equity.

### Consequences of #MeTooIndia Movement

The #MeToo movement in India catalyzed multiple discourses, revealing both systemic awareness and persistent structural inequities within the IEI [87,88]. Insights from study 2, based on in-depth interviews with scholarly experts, highlighted the movement’s mixed legacy—while it expanded public dialogue around sexual harassment, entrenched gender hierarchies and institutional practices remained largely unchanged [15,89]. This disconnect between awareness and reform mirrors broader societal resistance to redistributing power within patriarchal systems [87,90].

Participants described continuing patterns of victim blaming toward women who disclosed harassment, often amplified by media sensationalism. Such reactions perpetuate psychological distress, reputational harm, and social isolation, underscoring how hostile cultural responses can evolve into public health concerns through cumulative mental health and occupational stress [91,92]. Experts noted that survivors in high-visibility professions face compounded stigma and scrutiny, exacerbating trauma and discouraging disclosure. This outcome is consistent with global research on secondary victimization [22,38].

Interviewees also identified a persistent paradox in the IEI’s engagement with #MeToo, where increased public messaging on gender equity coexists with limited organizational accountability. This tension illustrates a symbolic commitment to feminist ideals rather than substantive institutional reform. Several experts described the movement’s momentum as short-lived “clicktivism” or “slacktivism,” characterized by digital enthusiasm but little lasting sociolegal impact [93-95]. Participants further noted that sustained solidarity was essential for continued engagement and resistance to social silencing, consistent with theories of collective action [96], though public disclosure frequently came with substantial psychological and professional costs.

Celebrities who spoke out faced coordinated online harassment, smear campaigns by industry elites, and defamation lawsuits aimed at intimidating or silencing them [16,89]. Such backlash reinforces patriarchal power structures and contributes to chronic distress, anxiety, and social withdrawal among women in high-visibility professions [20,92]. The hostile digital environment surrounding #MeTooIndia thus serves as an infoveillance signal of ongoing GBV and secondary victimization, illustrating how digital spaces can perpetuate trauma even while exposing it.

Despite these tensions, participants viewed the movement’s visibility as crucial for advancing public dialogue on sexual violence and strengthening institutional accountability. Achieving digital health and safety requires trauma-informed legal frameworks and industry mechanisms that protect survivors and translate online advocacy into sustainable, health-promoting reform. These findings should be interpreted alongside the limitations described in the next section.

### Limitations

Several key limitations influence the strength of the conclusions and recommendations drawn from this study. First, study 1 analyzed tweets collected over 1 month between November and December 2017, shortly after the initial #MeToo surge. This captures the early phase of the movement but may not represent later shifts in framing. Second, only English-language tweets were included, excluding parallel conversations in regional languages. Third, study 2 relied on 20 in-depth interviews with scholars and researchers specializing in gender, media, and film studies, constraining the analysis to the IEI context. While these experts provided valuable theoretical and contextual nuance, the perspectives of survivors (who experience digital hostility and secondary victimization directly) may differ in important ways. The absence of survivor interviews represents an additional limitation. Additionally, cultural norms, linguistic variations, and platform-specific affordances may have shaped the tone and visibility of #MeToo discourse, limiting the generalizability of findings across social and regional contexts.

### Conclusion

This study examined how social media discourse within the #MeToo movement reflects system-justifying narratives that sustain rather than dismantle existing hierarchies [5]. Contrary to prior studies emphasizing victim-blaming and overt backlash, findings from both study 1 and study 2 reveal strong evidence of gender and institutional system justification. These patterns indicate that social media often reinforces traditional gender norms and institutional authority, maintaining the status quo even within digital spaces that appear progressive. Although #MeToo expanded visibility and awareness, its ability to drive enduring structural change within sectors such as the IEI remains limited. Digital platforms, therefore, operate as contested arenas, where technologies that enable collective voice can simultaneously reproduce digital GBV.

Our findings extend scholarship that centers primarily on backlash and victim-blaming by demonstrating how broader system-justifying frames appear in social media discourses. The findings carry 3 major implications. First, for digital platforms, they highlight the need for proactive governance and content moderation policies that curb system-justifying retaliatory discourse. This requires integrating trauma-informed design principles to reduce secondary victimization. Second, for public health and policy stakeholders, the results point to the importance of recognizing digital hostility as a psychosocial health risk that warrants coordinated mental health and legal interventions. Third, for researchers and feminist advocates, this work offers an analytic lens for situating digital activism within broader institutional and psychological systems, while identifying points where transformative change may stall.

## Supplementary material

10.2196/75533 Multimedia Appendix 1 Semistructured interview guide for study 2 (#MeTooIndia IEI expert interviews). IEI: Indian Entertainment Industry.

10.2196/75533 Multimedia Appendix 2 Data and code files description: data_analysis_template.txt contains the Python code used for the computational framework, including preprocessing, sentiment analysis, stance detection, and topic modeling. ReadMe.md provides details about the dataset, file structure, and disclosure on data availability and usage. requirements.txt lists all Python package dependencies used in the analysis. sample_data_mt.txt includes a sample dataset excerpt.

10.2196/75533 Checklist 1 COREQ checklist.

## Abbreviations

GBV: gender-based violence
IEI: Indian Entertainment Industry
