# Supplementary material for: Digital Discourse, Secondary Victimization, and Psychological Harm: Mixed-Methods Analysis of System Justification in the #MeToo Movement
Source: J Med Internet Res. 2026 Apr 9;28:e75533. doi: 10.2196/75533 (PMC13067243; doi:10.2196/75533)
Supplement: Multimedia Appendix 1 [file jmir-v28-e75533-s001.docx]

**Semistructured Interview**

We conducted semistructured interviews using an interview guide developed from the insights of Study 1’s quantitative findings. The themes and justification categories identified through the Twitter analysis (gender, institutional, backlash, and victim-blaming justifications) informed the construction of open-ended questions aimed at exploring how these digital discourse patterns manifest within the Indian Entertainment Industry (IEI). This design allowed us to probe expert reflections on how online narratives correspond to, or diverge from, institutional and cultural realities in the post-#MeToo context.

**#MeTooIndia and IEI**

This interview explores expert perspectives on how the #MeTooIndia movement has influenced gender discourse, representation, and institutional practices within the Indian Entertainment Industry (IEI). The questions build upon findings from Study 1, which analyzed online narratives reinforcing or challenging system-justifying beliefs during #MeToo.

**Cultural and Institutional Impact**

- Since the #MeToo movement, what notable changes, if any, have you observed in the Indian Entertainment Industry (IEI)’s treatment of gender and sexual harassment?
- Do you think #MeToo has led to any lasting structural or cultural reforms within the industry?
- Do you believe #metoo campaign has been able to revolutionize IEI in any sense as opposed to earlier?

**Paradox of Representation**

- Several directors accused of harassment have continued to produce women-centered films. How do you interpret this paradox between their creative work and personal conduct?

**Public Persona vs. Private Behavior**

- Incidents such as the allegations against actor Alok Nath highlight the contradiction between on-screen moral portrayals and off-screen misconduct. How do you see this dynamic shaping public perception of gender norms and accountability in the Indian Entertainment Industry (IEI)?

**Digital Backlash and Victim-Blaming**

- Our Twitter analysis found persistent victim-blaming and backlash narratives. In your view, how has the #MeToo campaign in India confronted or reproduced these tendencies within media and public discourse?

**Shifts in Public and Media Sensitivity**

- Item numbers and sexist humor once normalized in the Indian Entertainment Industry (IEI) now often receive backlash. Do you see this as a sign of genuine social progress or temporary moral policing?

**Power and Hierarchies in the IEI**

- Economic and gender hierarchies make many women, especially, early-career artists vulnerable to exploitation. How do these structures perpetuate systemic silencing or enable justification of abuse?

**Normalization and Systemic Complicity**

- Figures such as Saroj Khan have publicly justified exploitation as “a part of the system.” Do such statements reflect internalized gender hierarchies and broader societal acceptance of inequality?

**Sustainability of Feminist Mobilization**

- Although #MeToo triggered nationwide debate, the momentum has waned. Do you think digital feminist movements can produce enduring transformation, or are they more symbolic expressions of temporary outrage?

**Closing Reflection**

- Based on your scholarship, what lessons can institutions, policymakers, or digital platforms draw from the #MeToo experience to foster safer and more equitable professional environments?
- And, last but not the least, as a scholar in this field, what would be your final comments?
