# Supplementary material for: Digital Discourse, Secondary Victimization, and Psychological Harm: Mixed-Methods Analysis of System Justification in the #MeToo Movement
Source: J Med Internet Res. 2026 Apr 9;28:e75533. doi: 10.2196/75533 (PMC13067243; doi:10.2196/75533)
Supplement: Checklist 1 [file jmir-v28-e75533-s003.pdf]

COREQ (COnsolidated criteria for REporting Qualitative research) Checklist

A checklist of items that should be included in reports of qualitative research. You must report the page number in your manuscript where you consider each of the items listed in this checklist. If you have not included this information, either revise your manuscript accordingly before submitting or note N/A.

Topic	Item No.	Guide Questions/Description	Reported on Page No.
**Domain 1: Research team and reflexivity**			
Personal characteristics			
Interviewer/facilitator	1	Which author/s conducted the interview or focus group?	1
Credentials	2	What were the researcher's credentials? E.g. PhD, MD	1
Occupation	3	What was their occupation at the time of the study?	1
Gender	4	Was the researcher male or female?	1
Experience and training	5	What experience or training did the researcher have?	1
Relationship with participants			
Relationship established	6	Was a relationship established prior to study commencement?	1
Participant knowledge of the interviewer	7	What did the participants know about the researcher? e.g. personal goals, reasons for doing the research	2
Interviewer characteristics	8	What characteristics were reported about the inter viewer/facilitator? e.g. Bias, assumptions, reasons and interests in the research topic	2
**Domain 2: Study design**			
Theoretical framework			
Methodological orientation and Theory	9	What methodological orientation was stated to underpin the study? e.g. grounded theory, discourse analysis, ethnography, phenomenology, content analysis	2
Participant selection			
Sampling	10	How were participants selected? e.g. purposive, convenience, consecutive, snowball	2
Method of approach	11	How were participants approached? e.g. face-to-face, telephone, mail, email	2
Sample size	12	How many participants were in the study?	3
Non-participation	13	How many people refused to participate or dropped out? Reasons?	3
Setting			
Setting of data collection	14	Where was the data collected? e.g. home, clinic, workplace	3
Presence of non-participants	15	Was anyone else present besides the participants and researchers?	3
Description of sample	16	What are the important characteristics of the sample? e.g. demographic data, date	3
Data collection			
Interview guide	17	Were questions, prompts, guides provided by the authors? Was it pilot tested?	3
Repeat interviews	18	Were repeat inter views carried out? If yes, how many?	3
Audio/visual recording	19	Did the research use audio or visual recording to collect the data?	3
Field notes	20	Were field notes made during and/or after the inter view or focus group?	3
Duration	21	What was the duration of the inter views or focus group?	4
Data saturation	22	Was data saturation discussed?	4
Transcripts returned	23	Were transcripts returned to participants for comment and/or	4

Topic	Item No.	Guide Questions/Description	Reported on Page No.
		correction?	
**Domain 3: analysis and findings**			
Data analysis			
Number of data coders	24	How many data coders coded the data?	4
Description of the coding tree	25	Did authors provide a description of the coding tree?	4
Derivation of themes	26	Were themes identified in advance or derived from the data?	4
Software	27	What software, if applicable, was used to manage the data?	4
Participant checking	28	Did participants provide feedback on the findings?	4
Reporting			
Quotations presented	29	Were participant quotations presented to illustrate the themes/findings? Was each quotation identified? e.g. participant number	4
Data and findings consistent	30	Was there consistency between the data presented and the findings?	5
Clarity of major themes	31	Were major themes clearly presented in the findings?	5
Clarity of minor themes	32	Is there a description of diverse cases or discussion of minor themes?	5

Developed from: Tong A, Sainsbury P, Craig J. Consolidated criteria for reporting qualitative research (COREQ): a 32-item checklist for interviews and focus groups. *International Journal for Quality in Health Care*. 2007. Volume 19, Number 6: pp. 349 – 357

Once you have completed this checklist, please save a copy and upload it as part of your submission. DO NOT include this checklist as part of the main manuscript document. It must be uploaded as a separate file.

COREQ (Consolidated Criteria for Reporting Qualitative Research) Checklist – Study 2

Manuscript Title: *Digital Discourse, Secondary Victimization, and Psychological Harm: A Mixed-Methods Analysis of System Justification in the #MeToo Movement*
Journal: *Journal of Medical Internet Research*
Manuscript Number: #75533
Study Component: *Study 2 – Qualitative Thematic Analysis*

Instructions:
This document contains complete responses to each COREQ item.

Domain 1: Research Team and Reflexivity

1 Which author/s conducted the interview or focus group?

The in-depth interviews were conducted by the joint first author-ST, who served as the primary interviewer for all synchronous and asynchronous interviews.

2 What were the researcher's credentials? E.g. PhD, MD

The interviewer was a doctoral researcher with advanced training in qualitative methods. Her academic background includes a BSc and MSc in Anthropology and an MA in Women and Gender Studies at the time of interviews.

3 What was their occupation at the time of the study?

At the time of the study, the interviewer (ST) was a doctoral student in the Department of Sociology at Louisiana State University.

4 Was the researcher male or female?

The interviewer (ST) identifies as female.

5 What experience or training did the researcher have?

The interviewer (ST) had eight years of experience conducting extensive ethnographic and other qualitative research. She published several qualitative manuscripts prior to this study and had received multiple fellowships and research grants supporting her qualitative work, including awards from the European Union and the Netherlands Research School of Gender Studies at Utrecht University, among others.

6 Was a relationship established prior to study commencement?

No prior relationship was established with the participants before the start of the study.

7. What did the participants know about the researcher? (e.g. personal goals, reasons for doing the research).

Participants were informed that the interviewer was an academic researcher conducting a qualitative study on gender, media, and the #MeToo movement in the Indian Entertainment Industry. They were given a general description of the study's purpose but were not informed of the specific analytical objectives or the prior quantitative study that informed the development of the interview guide. The semi-structured interviews were presented as an exploration of expert perspectives, and no additional personal goals or background information beyond the interviewer's academic role was shared.

8. What characteristics were reported about the interviewer/facilitator (e.g., bias, assumptions, reasons and interests in the research topic)?

The interviewer (ST) approached the study with an academic background in anthropology, gender studies, and qualitative inquiry. She maintained a reflexive stance throughout data collection, documenting analytic memos to minimize potential bias stemming from her scholarly interest in gender equity and digital discourse. No personal assumptions were shared with participants, and efforts were made to ensure that interview prompts remained open-ended and neutral.

Domain 2: Study design Theoretical framework

9 What methodological orientation was stated to underpin the study? e.g. grounded theory, discourse analysis, ethnography, phenomenology, content analysis

The study followed an inductive thematic analysis approach using the constant comparison method. This methodological orientation emphasized iterative coding, emergent theme development, and systematic comparison across transcripts to identify patterns in expert perspectives.

10 How were participants selected (e.g., purposive, convenience, consecutive, snowball)?

Purposive sampling was used to intentionally recruit academic experts whose scholarly work and disciplinary backgrounds made them well-positioned to provide informed perspectives on #MeTooIndia and the Indian Entertainment Industry (IEI).

11 How were participants approached? e.g. face-to-face, telephone, mail, email

Participants were approached through personalized email invitations that described the study and invited them to participate in an interview.

12 How many participants were in the study?

A total of 20 participants completed the study and formed the final qualitative sample.

13 How many people refused to participate or dropped out? Reasons?

Of the 29 individuals who responded to the invitation, 9 did not provide availability to schedule an interview; no additional refusals or dropouts were reported.

14 Where was the data collected (e.g., home, clinic, workplace)?

Data were collected through remote interviews, with synchronous interviews conducted via Zoom and asynchronous interviews conducted through email correspondence, allowing participants to engage from their personal or professional settings.

15 Was anyone else present besides the participants and researchers?

No individuals other than the participant and the interviewer were present during any interview.

16 What are the important characteristics of the sample (e.g., demographic data, date)?

The sample consisted of 20 academic experts in gender studies, media studies, and film studies affiliated with institutions in the United States, India, the United Kingdom, and Turkey. Demographic and professional characteristics are summarized in Table 3 of the manuscript.

17 Were questions, prompts, or guides provided by the authors? Was it pilot-tested?

A semi-structured interview guide was developed based on themes emerging from Study 1 and included open-ended questions covering gender narratives, institutional practices, digital backlash, and the #MeTooIndia context. The guide was reviewed for clarity but was not pilot tested prior to data collection.

18 Were repeat interviews carried out? If yes, how many?

No repeat interviews were conducted; however, asynchronous email interviews allowed follow-up questions when clarification or elaboration was needed.

19 Did the research use audio or visual recording to collect the data?

Synchronous interviews conducted via Zoom were audio-recorded with participant consent; asynchronous email interviews generated written records that served as transcript data.

20 Were field notes made during and/or after the interview or focus group?

Analytic memos and field notes were maintained during and after the interviews to support reflexivity and inform the thematic analysis.

21 What was the duration of the interviews or focus group?

Synchronous Zoom interviews typically lasted between 45 and 60 minutes and asynchronous email interviews unfolded over several exchanges across multiple days.

22 Was data saturation discussed?

Data collection concluded when thematic saturation was evident, as no new concepts emerged in the final interviews.

23 Were transcripts returned to participants for comment and/or correction?

Transcripts were not returned to participants for comment or correction.

Domain 3: analysis and findings

24 How many data coders coded the data?

Two researchers independently coded the transcripts during the initial coding phase to enhance analytic rigor.

25 Did authors provide a description of the coding tree?

A coding tree was developed iteratively during open and axial coding, with categories refined into thematic clusters reflecting gender system justification, institutional justification, victim blaming, and related subthemes.

26 Were themes identified in advance or derived from the data?

Themes were derived inductively from the data using an emergent, data-driven approach.

27 What software, if applicable, was used to manage the data?

NVivo 12 (QSR International) was used to organize transcripts, manage codes, and facilitate thematic clustering.

28 Did participants provide feedback on the findings?

Participants did not provide feedback on the findings.

29 *Were participant quotations presented to illustrate the themes/findings? Were quotations identified (e.g., participant number)?*

Quotations were included throughout the findings and attributed using pseudonyms to illustrate key themes and preserve participant confidentiality.

30 Was there consistency between the data presented and the findings?

Yes. The findings closely reflect the coded data, and thematic interpretations were supported with direct participant quotations.

31 Were major themes clearly presented in the findings?

Major themes which include gender system justification, victim blaming, and institutional system justification, were clearly described with supporting evidence.

32 Is there a description of diverse cases or discussion of minor themes?

Yes. The analysis includes examples reflecting diverse scholarly perspectives and nuances within themes, highlighting variation in interpretations and experiences.
